# Physical activity domains and risk of gastric adenocarcinoma in the MCC-Spain case-control study

**DOI:** 10.1371/journal.pone.0179731

**Published:** 2017-07-06

**Authors:** José M. Huerta, María Dolores Chirlaque, Antonio J. Molina, Pilar Amiano, Vicente Martín, Tania Fernández-Villa, Beatriz Pérez-Gómez, Víctor Moreno, Rosana Burgui, Inés Gómez-Acebo, Manuel Ramos-Lora, Guillermo Fernández-Tardón, Rosana Peiró, Rocío Olmedo-Requena, Marina Pollán, Manolis Kogevinas, Gemma Castaño-Vinyals, Nuria Aragonés, Navarro

**Affiliations:** 1 Department of Epidemiology, Murcia Regional Health Council, IMIB-Arrixaca, Murcia, Spain; 2 CIBER Epidemiología y Salud Pública (CIBERESP), Madrid, Spain; 3 Department of Health and Social Sciences, University of Murcia, Murcia, Spain; 4 Research Group on Gene-Environment Interactions and Health, University of León, León, Spain; 5 Public Health Division of Gipuzkoa, BioDonostia Research Institute, Government of the Basque Country, San Sebastián, Spain; 6 Cancer Epidemiology Unit, National Center for Epidemiology, Instituto de Salud Carlos III, Madrid, Spain; 7 Cancer Epidemiology Research Group, Oncology and Hematology Area, IIS Puerta de Hierro, Madrid, Spain; 8 Cancer Prevention and Control Program, Catalan Institute of Oncology- IDIBELL, L’Hospitalet de Llobregat, Spain; 9 Department of Clinical Sciences, Faculty of Medicine, University of Barcelona, Barcelona, Spain; 10 Navarra Public Health Institute, IdiSNA (Navarra Institute for Health Research), Pamplona, Spain; 11 Universidad de Cantabria-IDIVAL, Santander, Spain; 12 Department of Gastroenterology, Hospital Juan Ramón Jiménez, Huelva, Spain; 13 Centro de Investigación en Salud y Medio Ambiente (CYSMA), University of Huelva; 14 University of Oviedo-IUOPA, Oviedo, Spain; 15 Area de Cáncer y Salud Pública, Fundación FISABIO-Salud Pública, Valencia, Spain; 16 Department of Preventive Medicine and Public Health, University of Granada, Granada, Spain; 17 Instituto de Investigación Biosanitaria ibs, Granada, Complejo Hospitales Universitarios de Granada, University of Granada, Granada, Spain; 18 ISGlobal, Centre for Research in Environmental Epidemiology (CREAL), Barcelona, Spain; 19 IMIM (Hospital del Mar Medical Research Institute), Barcelona, Spain; 20 Universitat Pompeu Fabra (UPF), Barcelona, Spain; University of Oxford, UNITED KINGDOM

## Abstract

**Background:**

Evidence for a protective role of physical activity against development of stomach cancer is yet inconclusive. We studied the association of domain-specific physical activity and the risk of gastric adenocarcinoma (GAC), by site and histology, in the MCC-Spain case-control study.

**Methods:**

428 histologically confirmed GAC cases (67% men) including the gastro-esophageal region and 3225 controls were included. Cases were recruited in hospitals from 10 different Spanish regions, whereas population controls were randomly selected within the respective hospitals' catchment areas. A physical activity (PA) questionnaire was used to gather information on household and recreational activities, allowing estimation of PA volume (in metabolic equivalents (MET)-min/week). Participants also reported the intensity of working PA and daily sitting time. Questionnaire data on diet, lifestyles and clinical variables including *Helicobacter pylori* serology were available. Adjusted odds ratios (OR) of GAC were estimated for domains of physical activity, stratifying by sex, site (cardia vs. non-cardia), and Lauren classification (intestinal vs. diffuse).

**Results:**

Household physical activity (HPA) showed a strong inverse association with GAC, observed for both cardia and non-cardia tumours. Risk of overall gastric cancer was 50% lower risk among participants in the highest HPA category (OR = 0.50, 95%CI: 0.38, 0.66). Recreational physical activity (RPA) was also associated with lower overall GAC risk (OR = 0.68, 95% CI: 0.52, 0.88), particularly at moderate levels of intensity such as walking (OR = 0.61, 95% CI: 0.46, 0.79). The protective effect of RPA was strongest for non-cardia tumours. Sedentary time was not related to GAC risk (p-trend = 0.392), but the potential protective effect of RPA was restricted to non-sedentary participants.

**Conclusions:**

Both household and recreational physical activities were independently related to lower GAC risk in the MCC-Spain study.

## Introduction

Regular physical activity (PA) has been shown to reduce the risk of gastric cancer in observational studies. Previous meta-analyses suggest that people with a highly active lifestyle would benefit from a 10%-20% reduction in stomach cancer risk as compared to their least active counterparts [1–4]. Variations in risk estimates have been reported by sex (largest effect in women) [1–3], body mass index (weaker association at higher BMI) [2], smoking (weaker protection in smokers) [2], or tumour location [1–3,5]. However, these results are based on a limited number of observational studies, and the evidence to claim a preventive effect for PA against stomach cancer is still judged as insufficient [5–8].

Overall gastric cancer incidence has been decreasing for decades [9–11]. However, tumours of the cardia region are on a steady rise [11], and stomach cancer still represents the fifth most common cancer worldwide and the third leading cause of cancer-related mortality [12]. Furthermore, survival of gastric cancer patients is poor [13,14]. It is thus important to improve our knowledge of factors, such as PA, that may be effective in the primary prevention of the disease. Guidelines for cancer prevention [6] recommend doing a minimum of 150 minutes/week of moderate-intensity PA (or 75 minutes/week of vigorous-intensity PA) for a physically active lifestyle. However, it is yet unclear whether such recommended levels of PA could be effective in the prevention of gastric cancer, and the importance of domain-specific PA effects on health at different combinations of frequency and intensity should be further investigated [15].

The aim of this study was to gain insight into the association of domain-specific physical activity and gastric cancer risk by sex, tumour site, and histological type, in the MCC-Spain case-control study.

## Subjects and methods

### Study sample

The MCC-Spain project is a population multicase-control study carried out between September 2008 and December 2013 in 12 Spanish regions. Details on the study design, methods, and population characteristics have already been published [16]. Specifically, the sample for the present analysis included 428 newly diagnosed cases of stomach adenocarcinoma and 3264 population controls between 20 and 85 years old, recruited in 10 geographically dispersed provinces throughout Spain (Asturias, Barcelona, Cantabria, Granada, Huelva, León, Madrid, Murcia, Navarra, and Valencia).

Eligible patients were all gastric cancer cases with histological confirmation and no prior history of the disease, diagnosed within the study period (2008–2013) in the participating hospitals. The identification of cases was performed by active search through periodical visits to relevant hospital departments (i.e. gynaecology, urology, gastroenterology, oncology, general surgery, radiotherapy, and pathology departments). Confirmed gastric cancer cases with ICD-10 (International Classification of Diseases 10th Revision) codes C16 and D00.2 (carcinoma *in situ*) were recruited and interviewed as soon as possible after diagnosis. Simultaneously, common population controls were selected within the same hospitals' catchment areas of the cancer cases included in the MCC-Spain study. Control participants were randomly selected from the general practitioners lists, and frequency-matched to the pool of cancer cases (prostate, breast, oesophagus, stomach, and colon and rectum) by age, sex, and region. Because controls were not directly matched to gastric cancer cases (but to all cancer cases included in the MCC-Spain study), the analysis was not conditional on the matching. Controls from regions not contributing gastric cancer cases and those with previous history of the disease were not included in the analysis.

### Assessment of physical activity

A structured computerized epidemiological questionnaire was administered by trained personnel in a face-to-face interview. As for physical activity, detailed information on regular recreational activities was gathered for all participants in an open-ended manner. The questionnaire inquired about the type and frequency (in hours per week) of activities carried out for at least 6 months during the life course, registering the age at starting and (if applicable) the age at quitting the practice of each activity. Additionally, participants were also asked to report the number of weekly time dedicated to household activities of light intensity (*e*.*g*. cooking, washing dishes, ironing, making beds) and the total time (hours/week) invested in household tasks of higher intensity (*e*.*g*. scrubbing floors, washing windows, playing with children walking or running). Sedentariness was assessed by inquiring about the total hours/day spent sitting during leisure-time (including transportation), separately for weekdays and the weekend, and in reference to different time frames (the last year, the age period 30–39 years, and the age period 50–59 years if applicable). For occupational activity, participants were requested to report and classify every job they had had as: sedentary (almost exclusively sitting, without physical demand), low active (some physical demand, such as standing occupations or walking short distances), moderately active (manual work without manual handling of loads), quite active (physically demanding standing or walking occupations), or very active (vigorous occupations involving heavy energy expenditure).

Metabolic equivalent (MET) values were assigned to each reported recreational activity according to Ainsworth's Compendium of Physical Activities [17], 1 MET representing the rate of energy expenditure at resting state (defined as 1 kcal·kg^-1^·h^-1^). Weekly volume of recreational physical activity (in MET·min/week) was then computed for each participant as the sum of total time dedicated to each activity per week weighted by its corresponding MET value. Volume of household activities was computed likewise by assigning MET values of 2.8 and 3.5 to the categories of light, and higher intensity household activities, respectively. Sedentary time was obtained as the weighted mean of total hours/day spent sitting during weekdays and the weekend in the last year.

To minimise the potential of a reverse causation bias, occupational and recreational physical activity variables were defined to cover a ten-year exposure window up to the year previous to study entry (years -11 to -1, with recruitment being time 0), whenever possible. For occupational activity, participants were assigned the intensity category of their longest-lasting job within the exposure time frame, or the closest to the present, in case of a tie. When the sum of working years of a participant within the evaluated period was less than five, the participant was coded as 'not working'.

### Lifestyle data, anthropometry, and *Helicobacter pylori* infection status

The questionnaire further included questions on socio-demographic data, height, weight (of the previous year), family and personal medical history, drug use, reproductive history, smoking (one-year previous to cancer diagnosis or recruitment, in controls), and lifestyle habits. A socio-economic status score was developed as a combination of education, occupational social class, and self-reported parental socio-economic position. Diet of the previous year was assessed by means of a self-administered, validated, semi-quantitative food frequency questionnaire adapted for the MCC study by including additional items for regional products and cross-check questions [18]. Peripheral blood samples were drawn, and aliquoted fractions were stored at -80°C. Waist and hip circumferences were measured with an inelastic tape following standard procedures. Seroreactivity against a set of 15 *H*. *pylori* antigens was assessed by multiplex serology in serum samples, as detailed elsewhere [19]. Participants with reactivity for at least 4 out of the 15 *H*. *pylori* proteins tested were considered as positive for *H*. *pylori* infection.

### Exclusion criteria, classification of the exposure, and statistical analyses

For the present study exclusions affected non-adenocarcinoma gastric cancer cases (n = 31), and participants with missing information on physical activity variables (n = 215 controls). As for confounders, missing data were replaced by single imputation in continuous variables (<7% missingness), or by adding a 'missing' indicator category to factor variables. Dietary variables were categorised into quartiles, further adding a 'missing' category for participants who did not fill in the questionnaire (n = 561; 15.3%). The final sample available for analysis were 428 gastric adenocarcinoma (GAC) cases (n = 287 men), and 3225 controls. By site, there were 309 non-cardia GAC, 92 GAC of the esophagogastric junction, 14 adenocarcinoma of the lower third of the esophagus, and 13 cases with unspecified site or overlapping lesions. By histological type, as based on Lauren's criteria, tumours were classified as intestinal (n = 170), diffuse (n = 103), or mixed type (n = 20), whereas Lauren's type could not be determined for 135 cases based on the available data.

Physical activity variables were analysed according to specific domains: work, household, and recreational, plus sedentariness. Given that distribution of household activities largely differed by sex, the total volume of domestic activities (in MET·min/week) was categorised using sex-specific tertiles (labeled as 'lower', 'intermediate', and 'higher' household activity). For the recreational domain, *a priori* defined cut-offs were used based on general physical activity guidelines[20]. Leisure-time sitting time was categorised into 4 groups, per 3 hours/day increments.

Descriptive statistics performed were based on median values and inter-quartile ranges, for continuous variables, and frequencies (numbers and percentages) for categorical ones. Statistical differences between cases and controls were tested using Mann-Whitney *U* or χ^2^ tests, as appropriate.

Odds ratios (OR) and 95% confidence intervals (CI) of GAC by levels of physical activity were estimated using unconditional logistic regression models. The reference group was defined as the lowest category for physical activity variables, whereas the group spending <3 hours/day sitting was the referent for sedentariness. Multivariate models were adjusted for age, sex, socio-economic position (low, medium, high), study area, smoking (never, former, current), body mass index (in kg/m^2^), gastric symptomatology (no, yes, unknown), use of anti-inflammatory drugs (yes, no, unknown), family history of gastric cancer (no, yes, unknown), *H*. *pylori* serostatus (negative, positive, unknown), daily intake of total energy, red and processed meats, vegetables, and fruits, and past alcohol consumption (at participant's 30–40 years old), coded as low (<6 g/day in women or <12 g/day in men), moderate (women: 6–12 g/day; men: 12–24 g/day), and high (women: <12 g/day; men: >24 g/day). Tests for trend across physical activity categories were performed by assigning the median value to each level of household, recreational and sitting time variables, and including the variable as continuous in the regression model. For occupational activity, the trend was estimated by introducing the five-category variable as an ordinal predictor in the model, excluding non-workers. Heterogeneity in OR estimates for combinations of sedentariness and recreational physical activity across levels of selected factors (sex, age groups, socio-economic status, smoking, BMI, and infection by *H*. *pylori*) was assessed with likelihood-ratio tests comparing models with and without the interaction term.

All statistical analyses were performed with STATA/SE 12.1. *P*-values < 0.05 were considered statistically significant.

The writing of the manuscript followed the STROBE guidelines for reporting of observational studies (www.strobe-statement.org).

### Ethics statement

Participants who agreed to partake in the study signed an informed consent, and the protocol of MCC-Spain was approved by the local Ethics Committees of participating institutions (Comité Ético de Investigación Clínica (CEIC) del Instituto Municipal de Asistencia Sanitaria de Barcelona; CEIC del Hospital Universitario de Bellvitge; CEIC de Navarra; CEIC del Hospital Universitario La Paz; CEIC del Hospital Universitario Ramón y Cajal; CEIC de Cantabria; CEIC de la Dirección General de Salud Pública y Centro Superior de Investigación en Salud Pública; CEIC del Hospital General Universitario José Mª Morales Meseguer; Comité de Ética de la Investigación de la Provincia de Huelva; CEIC de León; Comité Ético de Investigación del Principado de Asturias; Comité de Ética de la Investigación Biomédica Provincial de Granada; Comité de Ética en Investigación Humana de la Universidad de Granada), in conformity to the principles of the Declaration of Helsinki. The database was registered in the Spanish Agency for Data Protection (no. 2102672171).

## Results

Median age at diagnosis was higher in cases than controls (68.8 vs. 65.8) (Table 1). Cases were predominantly men, had lower socio-economic status, were more likely overweight, and more frequently reported a family history of gastric cancer, and lower use of non-steroideal anti-inflammatory medication. Since men were over-represented among the cases, daily intake of energy, alcohol, and red meat were accordingly higher in this group (p < 0.001). For physical activity variables, significant differences by case status were found when comparing the proportion of participants regularly engaging in household (58.9% of cases vs. 76% of controls) or recreational activities (47.9% of cases vs. 60.1% of controls). However, median MET·min/week values did not differ between cases and controls who reported any amount of activity in these domains. Finally, no differences were found in sitting time between the two groups with a median of 5 hours/day of sitting time in both cases and controls.

**Table 1:** Descriptive characteristics of gastric adenocarcinoma cases and controls. MCC-Spain study.

	Controls		Cases		Non-cardia		Cardia		Intestinal		Diffuse		*P* cases vs. controls^1^
	(n = 3225)		(n = 428)		(n = 309)		(n = 106)		(n = 170)		(n = 103)		
Age	65.8	(16.4)	68.8	(19.6)	69.8	(19.0)	65.5	(20.2)	72.9	(14.6)	65.3	(21.5)	<0.001
Male sex	1759	(54.5)	287	(67.1)	185	(59.9)	94	(88.7)	112	(65.9)	53	(51.5)	<0.001
Low socio-economic status	1131	(35.1)	218	(50.9)	167	(54.1)	48	(45.3)	97	(57.1)	46	(44.7)	<0.001
Current smoker^2^	675	(20.9)	101	(23.6)	65	(21.0)	33	(31.1)	27	(15.9)	27	(26.2)	0.401
Overweight or obese^3^	2100	(65.1)	308	(72.0)	216	(69.9)	83	(78.3)	119	(70.0)	65	(63.1)	0.005
Family history of gastric cancer	356	(11.0)	90	(21.0)	71	(23.0)	16	(15.1)	45	(26.5)	25	(24.3)	<0.001
*Helicobacter pylori* seropositivity^4^	1777	(88.0)	239	(93.0)	176	(95.1)	56	(87.5)	97	(93.3)	56	(93.3)	0.017
NSAID medication	1250	(38.8)	116	(27.1)	79	(25.6)	33	(31.1)	40	(23.5)	31	(30.1)	<0.001
Energy intake (kcal/day)	1837	(743)	2027	(939)	1967	(922)	2156	(930)	1946	(955)	2094	(1149)	<0.001
Past alcohol consumption (g/day)	7.7	(24.3)	15.0	(42.7)	10.8	(35.9)	26.8	(52.7)	10.3	(44.0)	9.6	(32.8)	<0.001
Red meat intake (g/day)	56.2	(46.1)	71.8	(59.2)	66.3	(57.5)	86.6	(71.4)	72.4	(56.0)	67.0	(65.8)	<0.001
Fruit and vegetable intake (g/day)	517.2	(341.6)	517.9	(352.5)	513.0	(388.7)	503.5	(321.0)	530.4	(378.3)	534.7	(411.4)	0.711
Physical activity at work													
Sedentary or low active	990	(30.7)	112	(26.2)	71	(23.0)	37	(34.9)	35	(20.6)	32	(31.1)	0.144
Active or very active	491	(15.2)	66	(15.4)	44	(14.2)	20	(18.9)	18	(10.6)	23	(22.3)	
Not working	1744	(54.1)	250	(58.4)	194	(62.8)	49	(46.2)	117	(68.8)	48	(46.6)	
Household physical activity													
No	774	(24.0)	176	(41.1)	117	(37.9)	55	(51.9)	76	(44.7)	28	(27.2)	<0.001
MET-min/week^5^	2520	(3192)	2352	(3486)	2541	(3759)	1386	(2184)	2352	(3843)	2016	(3570)	0.182
Recreational physical activity													
No	1288	(39.9)	223	(52.1)	166	(53.7)	51	(48.1)	87	(51.2)	54	(52.4)	<0.001
MET-min/week^5^	1260	(1611)	1260	(1620)	1260	(1812)	1260	(1440)	1260	(1601)	1260	(1500)	0.413
Time spent sitting (h/day)	5.0	(4.6)	5.0	(4.0)	5.0	(4.0)	5.0	(5.0)	5.0	(3.7)	5.8	(4.0)	0.418

Values are medians and inter-quartile ranges for continuous variables, or numbers and percentages for categorical ones.

NSAID: Non-steroidal anti-inflammatory drugs.

^1^ P values from Mann-Whitney U or χ2 tests for the comparison of continuous and categorical variables, respectively, between cases and controls.

^2^ Smoking status in the previous year.

^3^ Body mass index calculated from self-reported weight in the previous year.

^4^ Data available for n = 2277 participants.

^5^ Among participants engaging in PA.

Multivariate logistic regression models of total GAC by physical activity domains showed highly significant inverse associations for household and recreational PA categories (Table 2). For the household domain, higher levels of PA were associated with lower OR of GAC by up to one half (OR = 0.50, 95% CI: 0.38–0.66; model 3), as compared to the lowest activity group ('none' for men, '<1000 MET·min/week' for women). Furthermore, participants who adhered to the recommended 500–1000 MET·min/week showed 35% lower risk of GAC (OR = 0.65, 95% CI: 0.43–0.98; model 3), as compared to those not engaging in RPA. On the other hand, associations were null for occupational activity and sedentary time. The significant results found were independent of all potential confounders considered (model 3), and each PA domain was further independent of all other domains evaluated (model 4). The strength of the association with RPA was slightly attenuated after accounting for BMI and dietary variables but remained, nonetheless, highly significant.

**Table 2:** Odds ratio (OR) and 95% confidence interval (CI) of gastric adenocarcinoma by levels of physical activity (PA) variables. MCC-Spain study.

		Model 1			Model 2			Model 3			Model 4		
	Controls / cases	OR	95% CI		OR	95% CI		OR	95% CI		OR	95% CI	
**Occupational PA**													
Sedentary or low active	990 / 112	1 (ref.)			1 (ref.)			1 (ref.)			1 (ref.)		
Active or very active	491 / 66	1.14	(0.82,	1.60)	1.10	(0.78,	1.54)	1.05	(0.74,	1.49)	1.10	(0.77,	1.56)
Not working^1^	1744 / 250	0.87	(0.64,	1.17)	0.84	(0.62,	1.14)	0.86	(0.63,	1.17)	0.85	(0.62,	1.16)
*P*_*trend*_		0.213			0.257			0.521			0.522		
**Household PA**													
Lower	875 / 191	1 (ref.)			1 (ref.)			1 (ref.)			1 (ref.)		
Intermediate	795 / 83	0.64	(0.47,	0.87)	0.62	(0.46,	0.84)	0.66	(0.48,	0.91)	0.66	(0.48,	0.91)
Higher	1391 / 127	0.48	(0.37,	0.63)	0.48	(0.37,	0.63)	0.50	(0.38,	0.66)	0.51	(0.39,	0.68)
*P*_*trend*_		< 0.001			< 0.001			< 0.001			< 0.001		
**Recreational PA (MET-min/week)**													
None	1288 / 223	1 (ref.)			1 (ref.)			1 (ref.)			1 (ref.)		
1–499	391 / 42	0.72	(0.50,	1.03)	0.73	(0.51,	1.06)	0.82	(0.56,	1.19)	0.82	(0.56,	1.19)
500–999	406 / 34	0.56	(0.38,	0.83)	0.58	(0.39,	0.86)	0.65	(0.43,	0.98)	0.67	(0.45,	1.02)
≥1000	1140 / 129	0.57	(0.44,	0.72)	0.58	(0.45,	0.75)	0.68	(0.52,	0.88)	0.73	(0.56,	0.95)
*P*_*trend*_		< 0.001			< 0.001			0.005			0.026		
**Sitting time (h/day)**													
<3	462 / 55	1 (ref.)			1 (ref.)			1 (ref.)			1 (ref.)		
3–5.9	1393 / 178	1.20	(0.86,	1.68)	1.12	(0.80,	1.58)	1.12	(0.79,	1.58)	1.07	(0.76,	1.52)
6–8.9	748 / 116	1.38	(0.96,	1.99)	1.27	(0.87,	1.83)	1.28	(0.88,	1.87)	1.25	(0.85,	1.83)
≥9	608 / 74	1.02	(0.68,	1.53)	0.94	(0.62,	1.42)	0.89	(0.58,	1.35)	0.78	(0.51,	1.19)
*P*_*trend*_		0.737			0.563			0.392			0.152		

Model 1: logistic regression adjusted for age, sex, socio-economic status, and study area.

Model 2: as model 1, plus further adjustment by smoking, presence of gastric symptomatology, use of anti-inflammatory drugs, family history of gastric cancer, and *Helicobacter pylori* seropositivity.

Model 3: as model 2, plus further adjustment by body mass index, and intake of total energy, red and processed meats, vegetables, and fruits, and past alcohol consumption.

Model 4: as model 3, but mutually adjusted by all physical activity domains in the table.

^1^ Nonworkers excluded from linear trend tests.

By site and histology, household PA (HPA) was found to be inversely associated with GAC irrespective of tumour location, but mainly of intestinal type (Table 3). On the other hand, RPA was more strongly associated with non-cardia GAC. The relationship of different variables for the household and recreational domains according to pre-specified levels of PA is presented in Table 4. Significant inverse linear trends were found for HPA or recreational walking (at any pace). RPA of moderate intensity was associated with lower GAC risk below the recommended 500 MET·min/week, whereas high-intensity RPA showed no relationship with to GAC risk overall. Finally, we tested for the combined effect of a sedentary behaviour (≥ 6 h/d sitting during leisure time) and adherence to PA recommendations (Table 5). Results revealed that recommended amounts of RPA were effectively associated with lower GAC risk, but only among non-sedentary participants. There was no evidence of effect modification by socio-demographic factors, smoking, BMI categories or *H*. *pylori* seropositivity. Although the protective association was mainly observed among men (Table 6), interactions with sex were not significant for any physical activity domain (p > 0.10).

**Table 3:** Odds ratio (OR) and 95% confidence interval (CI) of gastric adenocarcinoma, by site and type, according to levles of physical activity (PA) variables. MCC-Spain study.

	Non-cardia				Cardia				Intestinal				Diffuse			
	Cases	OR	95% CI		Cases	OR	95% CI		Cases	OR	95% CI		Cases	OR	95% CI	
**Occupational PA**																
Sedentary or low active	71	1 (ref.)			29	1 (ref.)			35	1 (ref.)			32	1 (ref.)		
Active or very active	44	1.08	(0.71,	1.63)	19	1.37	(0.73,	2.57)	18	0.91	(0.49,	1.68)	23	1.07	(0.60,	1.92)
Not working^1^	194	0.95	(0.66,	1.37)	44	0.66	(0.34,	1.25)	117	0.86	(0.52,	1.42)	48	0.65	(0.37,	1.16)
*P*_*trend*_		0.491				0.771				0.378				0.782		
**Household PA**																
Lower	129	1 (ref.)			50	1 (ref.)			86	1 (ref.)			33	1 (ref.)		
Intermediate	62	0.67	(0.46,	0.96)	17	0.63	(0.34,	1.18)	28	0.52	(0.31,	0.86)	26	0.86	(0.47,	1.54)
Higher	94	0.50	(0.37,	0.70)	23	0.42	(0.24,	0.73)	46	0.39	(0.25,	0.60)	36	0.68	(0.40,	1.17)
*P*_*trend*_		< 0.001				0.004				< 0.001				0.145		
**Recreational PA (MET-min/week)**																
None	166	1 (ref.)			48	1 (ref.)			87	1 (ref.)			54	1 (ref.)		
1–499	31	0.80	(0.52,	1.23)	6	0.59	(0.24,	1.44)	12	0.62	(0.32,	1.19)	14	1.05	(0.55,	1.98)
500–999	25	0.62	(0.39,	0.99)	6	0.58	(0.24,	1.42)	15	0.74	(0.40,	1.37)	8	0.58	(0.26,	1.27)
≥1000	87	0.60	(0.44,	0.81)	32	0.77	(0.47,	1.28)	56	0.67	(0.45,	1.00)	27	0.61	(0.37,	1.02)
*P*_*trend*_		0.001				0.427				0.085				0.049		
**Sitting time (h/day)**																
<3	33	1 (ref.)			16	1 (ref.)			22	1 (ref.)			15	1 (ref.)		
3–5.9	139	1.50	(0.98,	2.27)	32	0.61	(0.31,	1.17)	77	1.32	(0.77,	2.24)	38	0.95	(0.50,	1.81)
6–8.9	83	1.59	(1.01,	2.51)	23	0.73	(0.35,	1.50)	48	1.54	(0.86,	2.75)	27	1.24	(0.62,	2.46)
≥9	52	1.02	(0.61,	1.69)	19	0.68	(0.31,	1.48)	22	0.77	(0.38,	1.55)	22	1.04	(0.50,	2.16)
*P*_*trend*_		0.309				0.898				0.243				0.719		

Odds ratios from logistic regression models adjusted for age, sex, socio-economic status, study area, smoking, body mass index, gastric symptomatology, use of anti-inflammatory drugs, family history of gastric cancer, *Helicobacter pylori* seropositivity, intake of total energy, red and processed meats, vegetables, and fruits, and past alcohol consumption.

^1^ Nonworkers excluded from linear trend tests.

**Table 4:** Odds ratio (OR) and 95% confidence interval (CI) of gastric adenocarcinoma, by intensity of household and recreational physical activity variables. MCC-Spain study.

	Categories of physical activity							*P*_*trend*_
**Household, lower intensity**	None (men) / <1 h/d (women)	<1 h/d (men) / 1–3 h/d (women)			≥1 h/d (men) / ≥3 h/d (women)			
Controls / cases	1048 / 215	1002 / 92			1102 / 108			
OR (95% CI)	1 (ref.)	0.61	(0.46,	0.81)	0.61	(0.46,	0.82)	< 0.001
**Household, higher intensity**	None (men) / <1 h/d (women)	<1 h/d (men) / 1–3 h/d (women)			≥1 h/d (men) / ≥3 h/d (women)			
Controls / cases	2019 / 292		773 / 76		279 / 33			
OR (95% CI)	1 (ref.)	0.78	(0.58,	1.05)	0.62	(0.39,	0.98)	0.017
**Walking**	None	1–500 MET-min/week			≥500 MET-h/week			
Controls / cases	1961 / 301		283 / 29		981 / 98			
OR (95% CI)	1 (ref.)	0.77	(0.50,	1.19)	0.61	(0.46,	0.79)	< 0.001
**Recreational, moderate intensity (walking excluded)**	None	1–500 MET-min/week			≥500 MET-min/week			
Controls / cases	2190 / 335		363 / 23		665 / 69			
OR (95% CI)	1 (ref.)	0.54	(0.34,	0.85)	0.73	(0.54,	0.98)	0.124
**Recreational, high intensity**	None	1–500 MET-min/week			≥500 MET-min/week			
Controls / cases	3066 / 406		41 / 6		117 / 16			
OR (95% CI)	1 (ref.)	1.23	(0.49,	3.08)	1.20	(0.67,	2.14)	0.770

HPA: Household physical activity; RPA: recreational physical activity.

Odds ratios from logistic regression models adjusted for age, socio-economic status, study area, smoking, body mass index, gastric symptomatology, use of anti-inflammatory drugs, family history of gastric cancer, *Helicobacter pylori* seropositivity, intake of total energy, red and processed meats, vegetables, and fruits, and past alcohol consumption.

**Table 5:** Odds ratio (OR) and 95% confidence interval (CI) of gastric adenocarcinoma by combined levels of sedentary behaviour and recreational physical activity (RPA) recommendations. MCC-Spain study.

			Sedentary /	Non-sedentary /			Sedentary /			Non-sedentary /			
			insufficient RPA^1^	insufficient RPA			sufficient RPA			sufficient RPA			
		Controls / cases	785 / 115	884 / 14			571 / 75			971 / 86			*P*_interaction_
All		3225 / 428	1 (ref.)	1.24	(0.92,	1.67)	0.93	(0.66,	1.30)	0.68	(0.48,	0.94)	
Sex	Men	1759 / 287	1 (ref.)	1.18	(0.81,	1.73)	0.75	(0.49,	1.13)	0.57	(0.38,	0.86)	0.113
	Women	1466 / 141	1 (ref.)	1.56	(0.91,	2.65)	1.49	(0.79,	2.83)	1.02	(0.55,	1.91)	
Age	<65 years	1501 / 161	1 (ref.)	1.45	(0.91,	2.31)	0.95	(0.52,	1.71)	0.62	(0.35,	1.11)	0.826
	≥65 years	1724 / 267	1 (ref.)	0.99	(0.67,	1.47)	0.91	(0.60,	1.38)	0.60	(0.39,	0.91)	
Socio-economic status	Low	1131 / 218	1 (ref.)	1.47	(0.94,	2.30)	1.10	(0.65,	1.86)	0.66	(0.40,	1.09)	0.441
	Medium or high	2094 / 210	1 (ref.)	1.13	(0.74,	1.71)	0.82	(0.52,	1.29)	0.73	(0.46,	1.15)	
Smoking habit	Never	1421 / 174	1 (ref.)	1.47	(0.89,	2.42)	1.29	(0.73,	2.28)	0.79	(0.45,	1.40)	0.835
	Former	1113 / 152	1 (ref.)	1.28	(0.74,	2.23)	0.84	(0.48,	1.48)	0.59	(0.33,	1.04)	
	Current	675 / 101	1 (ref.)	0.98	(0.54,	1.78)	0.66	(0.29,	1.48)	0.74	(0.37,	1.50)	
Body mass index	Normal weight	1091 / 117	1 (ref.)	1.75	(0.95,	3.23)	1.16	(0.57,	2.37)	0.73	(0.36,	1.45)	0.493
	Overweight/obese	2100 / 308	1 (ref.)	1.13	(0.79,	1.60)	0.88	(0.60,	1.30)	0.70	(0.47,	1.03)	
*H*. *pylori* seropositivity	No	243 / 18	1 (ref.)	0.47	(0.06,	3.48)	0.44	(0.04,	4.51)	0.18	(0.02,	1.65)	0.248
	Yes	1777 / 239	1 (ref.)	1.18	(0.80,	1.73)	0.78	(0.49,	1.24)	0.55	(0.35,	0.85)	

^1^ A sedentary behaviour was defined as spending 6 or more h/d of leisure time sitting. Recreational physical activity (RPA) was considered insufficient when the sum of all recreational activities did not meet the recommended 500 MET-minutes/week, and was considered sufficient otherwise.

Odds ratios from logistic regression models adjusted for age, sex, socio-economic status, study area, smoking, body mass index, gastric symptomatology, use of anti-inflammatory drugs, family history of gastric cancer, *Helicobacter pylori* seropositivity, intake of total energy, red and processed meats, vegetables, and fruits, and past alcohol consumption.

**Table 6:** Odds ratio (OR) and 95% confidence interval (CI) of gastric adenocarcinoma by sex, according to levels of physical activity (PA) variables. MCC-Spain study.

	All				*Helicobacter pylori* positive			
	Controls / cases	OR	95% CI		Controls / cases	OR	95% CI	
**MEN**								
Occupational PA								
Sedentary or low active	564 / 88	1 (ref.)			318 / 49	1 (ref.)		
Active or very active	288 / 47	1.08	(0.71,	1.65)	152 / 25	1.04	(0.58,	1.86)
Not working^1^	907 / 152	0.91	(0.61,	1.35)	538 / 93	0.95	(0.57,	1.60)
*P*_*trend*_		0.642				0.212		
Household PA (MET-min/week)								
None	728 /171	1 (ref.)			443 / 101	1 (ref.)		
1–999	333 / 42	0.59	(0.39,	0.88)	184 / 21	0.57	(0.33,	0.99)
≥1000	617 / /65	0.41	(0.29,	0.58)	337 / 39	0.51	(0.33,	0.78)
*P*_*trend*_		< 0.001				0.003		
Recreational PA (MET-min/week)								
None	672 / 155	1 (ref.)			382 / 97	1 (ref.)		
1–499	165 / 24	0.73	(0.44,	1.20)	99 / 14	0.61	(0.32,	1.17)
500–999	210 / 18	0.51	(0.30,	0.89)	122 / 7	0.27	(0.12,	0.63)
≥1000	712 / 90	0.57	(0.42,	0.79)	405 / 49	0.51	(0.34,	0.78)
*P*_*trend*_		0.001				0.004		
Sitting time (h/day)								
<3	209 / 36	1 (ref.)			132 / 27	1 (ref.)		
3–5.9	741 / 112	0.91	(0.58,	1.43)	436 / 59	0.65	(0.37,	1.13)
6–8.9	450 / 80	1.14	(0.70,	1.85)	253 / 50	0.96	(0.53,	1.73)
≥9	353 / 54	0.77	(0.46,	1.32)	184 / 31	0.67	(0.34,	1.32)
*P*_*trend*_		0.429				0.695		
**WOMEN**								
Occupational PA								
Sedentary or low active	426 / 24	1 (ref.)			207 / 13	1 (ref.)		
Active or very active	203 / 19	1.05	(0.53,	2.11)	122 / 9	0.70	(0.26,	1.91)
Not working^1^	837 / 98	0.86	(0.48,	1.54)	440 / 50	0.99	(0.43,	2.27)
*P*_*trend*_		0.977				0.488		
Household PA (MET-min/week)								
<1000	147 / 20	1 (ref.)			76 / 15	1 (ref.)		
1000–2999	462 / 41	0.81	(0.41,	1.58)	221 / 17	0.57	(0.23,	1.42)
≥3000	774 / 62	0.66	(0.35,	1.25)	423 / 34	0.49	(0.22,	1.10)
*P*_*trend*_		0.207				0.161		
Recreational PA (MET-min/week)								
None	616 / 68	1 (ref.)			322 / 34	1 (ref.)		
1–499	226 / 18	1.02	(0.55,	1.91)	118 / 12	0.92	(0.40,	2.11)
500–999	196 / 16	0.85	(0.44,	1.66)	91 / 6	0.73	(0.26,	2.08)
≥1000	428 / 39	0.95	(0.58,	1.57)	238 / 20	0.90	(0.44,	1.83)
*P*_*trend*_		0.836				0.805		
Sitting time (h/day)								
<3	253 / 19	1 (ref.)			142 / 14	1 (ref.)		
3–5.9	652 / 66	1.62	(0.89,	2.96)	334 / 33	0.90	(0.41,	2.00)
6–8.9	298 / 36	1.53	(0.79,	2.98)	127 / 16	1.36	(0.55,	3.38)
≥9	255 / 20	0.94	(0.44,	2.02)	164 / 9	0.41	(0.13,	1.23)
*P*_*trend*_		0.354				0.146		

Odds ratios from logistic regression models adjusted for age, socio-economic status, study area, smoking, body mass index, gastric symptomatology, use of anti-inflammatory drugs, family history of gastric cancer, *Helicobacter pylori* seropositivity, intake of total energy, red and processed meats, vegetables, and fruits, and past alcohol consumption.

^1^ Non workers excluded from linear trend tests.

## Discussion

Household and recreational physical activities were strongly associated with a lower risk of GAC in the MCC-Spain study. The associations found were independent of a wide set of potential confounders and other physical activity domains. In stratified analysis, associations were highly consistent for non-cardia tumours, and mainly among men, although interactions with sex were not significant. On the other hand, sedentary time was not found to be a risk factor for gastric cancer in this study.

The promotion of physical activity is acknowledged as one of the hallmarks of chronic disease prevention, and an important contributor to wellbeing and healthy ageing [6,7,21]. In order to achieve such goals, guidelines recommend spending at least 150 min/week in activities of moderate intensity (or equivalently 75 min of high-intensity PA). Expressed as volume, this would represent 500–1000 MET·minutes/week of moderate-intensity PA (or an equivalent volume of high-intensity activities), which is advised to adults and older people with no limiting conditions [20,21]. While guidelines encourage people to remain active in all domains of everyday life, many times practical indications or specific recommendations to the population are formulated in terms of recreational outdoor activities. However, in the epidemiological literature there is also support for a protective effect of non-recreational PA as determinant of chronic disease risk. Our results revealed a strong association for household PA, with the lowest GAC risks among all PA variables observed for household PA (50% lower risk for the 'highest' *versus* 'lowest' group comparison). The strong inverse association of HPA with chronic disease adds to the accruing epidemiological evidence showing decreased risks of cancer and overall mortality at increasing levels of HPA[22–24]. A previous meta-analysis [23] found that overall risk of mortality was reduced by 36% (RR = 0.64, 95% CI: 0.55–0.75; p = 0.039) when comparing the highest and lowest groups of activities of daily living (household plus walking and cycling). In a more recent dose-response meta-analysis of HPA and cancer risk, Shi *et al*. [22] described an inverse linear relationship, with an estimated RR = 0.84 (95% CI: 0.76, 0.93) for the highest vs. lowest comparison, and 2% risk reduction per additional 10 MET·h/week. Shi *et al*.'s meta-analysis provides suggestive evidence for a protective effect of HPA in cancer risk. However, such association was mainly dominated by studies on PA and breast cancer. Unfortunately, only two studies on gastric cancer were available for meta-analysis (1 cohort [25] and 1 case-control [26]), both reporting null results. However, the first one did not estimate separated effects for HPA, whereas the assessment of HPA in Wen *et al*.'s paper was not quantitative (furthermore, PA was not the main exposure in the study and multivariate models lacked important confounders such as education, *H*. *pylori* infection, or red and processed meat intake). Thus, this is the first study to provide specific quantitative estimates of the association of HPA of different intensities and gastric cancer risk, with extensive adjustment for relevant confounders (demographics, diet, lifestyles, family history, medication, or *H*. *pylori* serostatus). Further studies are needed to corroborate our results.

Household PA comprises a plethora of indoor and outdoor activities of light, moderate, or intense effort that actually represents the largest PA domain for different population groups. In the present study, HPA accounted for 83% of total (non-occupational) activity volume, on average, among women, and for more than half total non-occupational activity in men below 60 years old. Such heterogeneity in HPA allowed for more powerful comparisions across exposure categories which could partly explain the larger effect estimates found for HPA as compared to recreational activities. As expected, men and women had very different distributions of HPA (only 3% of women reported no HPA at all vs. 43% of men). Thus, sex-specific tertiles were defined to preclude a severe imbalance of the sex ratio among HPA groups. However, the 'low HPA' group in women covered a PA range (< 1000 MET·minutes/week) for which effects in men were significant and strong (Table 6). The lack of an appropriate reference group of women not engaging in HPA, and the reduced statistical power due to the fewer number of cases, would mostly explain the null associations found in this group, as very few women had HPA levels low enough as to allow for powered statistical comparisons. Nevertheless, since there was no statistical evidence of effect modification by sex (*p* for sex interaction = 0.306), no specific conclusions should be drawn from sex-specific analyses. Rather, the accumulation of data supporting a protective effect of HPA on cancer and chronic disease risk in cross-sectional and longitudinal studies, advocates not to neglect the household dimension of physical activity either when conducting epidemiological research or when translating study results into recommendations or interventions for the population.

Associations of recreational PA with GAC were highly significant within the recommended 500–1000 MET·min/week range, with an estimated OR (95% CI) of 0.65 (0.43, 0.98). It is noteworthy that moderate RPA (but not walking or high-intensity activities) was associated with lower GAC risk even at levels below such recommendation (OR = 0.54, 95% CI: 0.34, 0.85). Previous meta-analyses of case-control studies found slightly lower OR for sufficient vs. insufficient (OR = 0.78, 95% CI: 0.66, 0.91) [2] or high vs. low levels of RPA (OR from 0.75 to 0.84) [1,3,4], while pooled estimates from cohort studies were of lower magnitude (OR from 0.81 to 0.87) [1–4]. Our results suggest that prevention of GAC through RPA should mainly rely on the promotion of moderate-intensity activities (such as walking, bicycling, swimming, or home exercise) rather than on RPA of higher intensity, for which we found null results. Of note, Behrens *et al*. [1] described a curvilinear association for RPA and gastroesophageal cancer with the lowest risk at 5 times/week of moderate-to-high RPA, and non-significant risk reductions at increased frequency. Thus, while our data support moderating the intensity of RPA to get the maximal benefit, others have suggested moderating the frequency of PA for optimal prevention of gastric cancer.

The question as to whether PA effects would differ by tumour site or histological type of GAC remains elusive. Our data support previous results from large-scale prospective studies [25,27] showing larger effects of RPA on distal (non-cardia) tumours. However, a recent report by Moore *et al*. [5] using pooled prospective data from 1.44 million participants showed no significant association overall for non-cardia gastric cancer (HR = 0.92, 95% CI: 0.81, 1.06), whereas the significant association reported for cardia tumours was shown to be largely mediated by BMI. It is important to note that despite the large number of subjects, standardised analytical methodology, and prospective nature of data included in Moore's dataset, the number of studies used for estimation of GC risk was low (n = 7), and thus further research is warranted to be able to draw more definitive conclusions on this topic. Even fewer investigations have considered a potential heterogeneity by histology of GAC. We have found the effect of HPA to be only significantly associated with tumours of intestinal type, while for recreational PA, similarly borderline associations were found for intestinal or diffuse types among participants in the upper vs. lower PA categories (Table 3). The paper by Moore *et al*. did not report on the effect of RPA on histological subtypes of gastric cancer since papers addressing the study of such association are very scarce. In a previous paper with prospective data from the EPIC study [25], no significant associations in GAC risk by histological sub-types were reported, although the number of cases was also low for sub-group analyses, limiting the statistical power. To our knowledge, no other epidemiological study has addressed this association. Due to the scarcity of data, further studies with a sufficient number of cases are needed that analyse the specific effect of PA by histological sub-types of GAC.

Sitting time was not associated with GAC overall or in sub-group analyses by site or histology. Associations were null also when considering working days and weekends separately (data not shown). There is only one previous paper, from the NIH-AARP Diet and Health Study [27], that looked at sedentary time in relation to gastric cancer risk, which reported similar null findings either for television watching or daily sitting time. A sedentary behaviour has been estimated to account for 10% of colon cancers and 10% of breast cancers globally [28], but the evidence so far does not support an independent effect on stomach cancer risk. Nevertheless, cross-classification of subjects according to sedentary time and level of RPA in the present study revealed that RPA was only associated with GAC among non-sedentary participants (Table 5). This finding suggests that physical inactivity may be able to counterbalance the benefits of physical activity on chronic disease risk (as supported by previous data on HPA and premature mortality [29]), and reinforces the message that active lifestyles cannot be defined based solely on the amount of PA, but also on limiting sedentary behaviours.

The present study has some limitations. Case-control studies are prone to recall bias and potential reverse causation. In the present study, cases were recruited as close to the date of diagnosis as possible, with over 76% of GAC cases interviewed within three months of cancer diagnosis. Since pre-diagnostic conditions might affect exposure assessment, data were collected excluding recent exposure whenever possible. Thus, recreational physical activity was assessed using an open-ended questionnaire that recorded age at starting (and quitting) of every activity reported, which allowed to exclude exposures within the last year previous to study entry. Diet, smoking status and estimated BMI were also defined for the previous year, and alcohol intake was referred to consumption in the 30–40 year-old period (over 97% of cases were diagnosed after the age of 40 years). However, reverse causation cannot be totally discarded for HPA or sitting time variables, meant to reflect the habitual engagement in such activities on a mid-term basis, but that could have been partly affected by pre-diagnostic conditions among the cases. Nevertheless, it is unlikely that reverse causation is a major explanation for our findings, since there is increasing evidence from prospective studies with long follow-up (thus, free of reverse causation bias) supporting a protective role for household activities against risk of chronic disease and mortality. Another concern is the scarcity of cases among certain strata of tumour location (*e*.*g*. cardia) or histology (*e*.*g*. diffuse type) that may have limited the statistical power to detect potential associations. Confounding (either residual or unmeasured) is always an issue in the analysis of observational data. Because of the multicase-control design of the study, controls were matched to the whole pool of cancer cases, and the separate analysis of individual cancer locations (*e*.*g*. stomach) limits the efficiency of the matching procedure. Thus, all models included matching factors (age, sex, and region) as covariates. Furthermore, the final models included the most relevant potential confounders, plus other important factors, such as *H*. *pylori* serostatus, not available to adjust for in most previous epidemiological studies. Unfortunately, the sensitive approach used to define *H*. *pylori* seropositivity did not allow us to stratify results on *H*. *pylori* serostatus, and stratified results are only presented for positive cases (Table 6). Finally, selection bias was addressed by aiming to recruit all cancer cases diagnosed within the study period in the selected areas, and the use of population controls selected from general practitioners lists. However, the non-universal sampling frame of controls and the low response rate (57% for gastric cancer patients) [16] may account for some degree of selection bias, which would limit the generalizability of our results.

Strengths of the study include the availability of data on gastric cancer site and histology to allow for sub-group analyses, the large set of confounders available to adjust for, including *H*. *pylori* infection, and the exhaustive, open-ended, PA questionnaire that included specific questions on the main PA domains (work, household, and recreational) and sedentary time.

## Conclusion

Our results support a protective role for PA on gastric cancer risk, particularly non-cardia tumours, with the largest effects found for household PA. Recommended levels of recreational activity were significantly associated with lower GAC risk, but only among non-sedentary participants. Our data advice promoting PA in all domains of everyday life, not neglecting household activities, and concomitantly limiting sitting time. Such recommendations, as formulated in the 4th European Code Against Cancer [6], should be the framework for effective public health action.

## Supporting information

S1 Fig Odds ratios of gastric cancer by levels of non-occupational physical activity (household plus recreational PA) across sitting time categories in the MCC-Spain study. Range (in MET·minutes/week) of non-occupational PA categories: T1: 1–1380 (men), 1–3150 (women), T2: 1381–3105 (men), 3151–5460 (women), T3: >3105 (men), >5460 (women).
(TIF)
