# Correction: Physical activity domains and risk of gastric adenocarcinoma in the MCC-Spain case-control study

**DOI:** 10.1371/journal.pone.0184460

**Published:** 2017-08-31

**Authors:** The PLOS ONE Staff

The final author's name is incorrect. The correct name is: Carmen Navarro. The publisher apologizes for the error.
