# Supplementary figures and images for: Physical activity domains and risk of gastric adenocarcinoma in the MCC-Spain case-control study
Source: PLoS One. 2017 Jul 6;12(7):e0179731. doi: 10.1371/journal.pone.0179731 (PMC5500262; doi:10.1371/journal.pone.0179731)

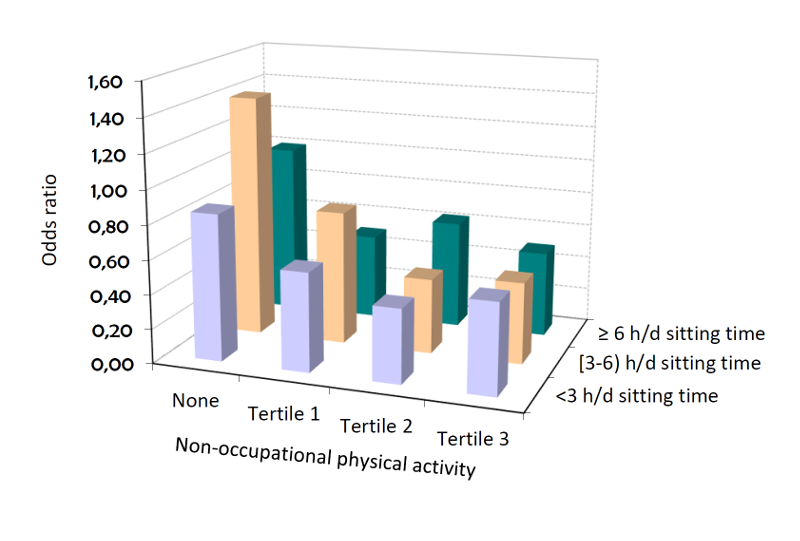

Supplement: S1 Fig — Range (in MET·minutes/week) of non-occupational PA categories: T1: 1–1380 (men), 1–3150 (women), T2: 1381–3105 (men), 3151–5460 (women), T3: >3105 (men), >5460 (women). (TIF) [file pone.0179731.s001.tif]
